# A COTS-Based Portable System to Conduct Accurate Substance Concentration Measurements

**DOI:** 10.3390/s18020539

**Published:** 2018-02-10

**Authors:** Juan Aznar-Poveda, Jose Antonio Lopez-Pastor, Antonio-Javier Garcia-Sanchez, Joan Garcia-Haro, Toribio Fernández Otero

**Affiliations:** 1 Department of Information and Communication Technologies (TIC), Technical University of Cartagena, ETSIT, Campus Muralla del Mar, E-30202 Cartagena, Spain; juan.aznarp@gmail.com (J.A.-P.); joseantonio.lopez@upct.es (J.A.L.-P.); joang.haro@upct.es (J.G.-H.); 2 Center for Electrochemistry and Intelligent Materials (CEMI), Technical University of Cartagena, ETSII, Campus Alfonso XIII, E-30203 Cartagena, Spain; toribio.fotero@upct.es

**Keywords:** screen-printed electrode, potentiostat, cyclic voltammetry

## Abstract

Traditionally, electrochemical procedures aimed at determining substance concentrations have required a costly and cumbersome laboratory environment. Specialized equipment and personnel obtain precise results under complex and time-consuming settings. Innovative electrochemical-based sensors are emerging to alleviate this difficulty. However, they are generally scarce, proprietary hardware and/or software, and focused only on measuring a restricted range of substances. In this paper, we propose a portable, flexible, low-cost system, built from commercial off-the-shelf components and easily controlled, using open-source software. The system is completed with a wireless module, which enables the transmission of measurements to a remote database for their later processing. A well-known PGSTAT100 Autolab device is employed to validate the effectiveness of our proposal. To this end, we select ascorbic acid as the substance under consideration, evaluating the reliability figure and obtaining the calibration curves for both platforms. The final outcomes are shown to be feasible, accurate, and repeatable.

## 1. Introduction

Advances in accurate substance concentration measurements depend on the progress of new devices/systems, which apply emerging technologies. Traditional procedures and methods involve the use of complex laboratory equipment and exhaustive control of multiple external variables, as with high performance liquid chromatography (HPLC) [1] or spectrophotometry [2], among others. They take longer and require more effort to develop specific treatments and processes aimed at obtaining appropriate samples for further analysis. Despite being the most popular methods in areas as diverse as water quality [3], food safety [4], medical diagnosis [5], or drug control [6], their high cost and the need for specialized staff and proprietary licenses restrict their use to big companies, public institutions, or other similar organizations. Furthermore, the size of the equipment limits portability, making it, therefore, advisable to install these systems in specialized laboratories.

Recent developments in sensors based on electrochemical techniques [7,8,9,10] and their associated electronics have favorably impacted on this research field, since they offer appreciable advantages over the laboratory procedures. Many innovative biosensor types, such as nanowire arrays, FET devices or three-electrode systems are emerging. The latter are the most widely used for their simplicity. Three-electrode sensors become key components of the so-called points-of-care (henceforth, PoC). A PoC is a small, portable device, developed principally by the pharmaceutical industry, capable of determining very low levels of given substances (e.g., proteins) such as glucose [11]. Its purpose is to avoid inconvenient displacements to perform blood tests or other analyses. In addition, the latest advances also allow the delivering of test results through a mobile network [12]. Other areas, such as food quality control, are also interested in similar devices. So, in [13], a miniature PoC-like instrument that includes hand-made electrodes of graphite, accurately measures, “in situ” (e.g., in a crop) concentrations of substances such as vitamins, acids or carbohydrates present in fruit, vegetables, cereals and beverages, and can indicate whether the product fulfills the expected quality. In other cases, the aforementioned electrodes are printed on ceramics, and are denoted screen-printed electrodes (hereinafter referred to as SPE). These electrodes are a well-suited solution due to their low-cost, easy production, use, maintenance, disposability and handling. Diverse companies such as Metrohm-Autolab B.V. [14], DropSens [15] or PalmSens [16], provide both SPE and the measuring instrument, called potentiostat, within their respective product catalogs. 

Nowadays, these systems are proprietary. They are only able to conduct tests for a small number of specific substances, and are not affordable for the general public due to their cost. A more flexible solution is therefore necessary, to fully satisfy a higher number of target customers, while keeping costs low and avoiding proprietary designs. In this sense, there are recent projects intended to design open-source and highly accurate potentiostats, though they are conceived for non-portable laboratory environments [17,18].

To overcome this, diverse portable and open-source potentiostat prototypes are being developed. So, in the education field, we find the proposal in [19]—a straightforward device mainly focused on teaching students. It lacks wireless communication interfaces and a formal validation studio is not provided. In [20,21], multimodal electrochemical methods are implemented for different applications using complex and custom-made sensors; such as improving the specificity of ethanol detection in multiple analyte and low concentration solutions. Furthermore, in these works, users must acquire specific skills, in order to operate the equipment. From the design of the silicon board to the acquisition and welding of the required components the entire electronics must be implemented, which clearly reduces their reproducibility. To face these drawbacks, commercial-off-the-shelf (COTS) components should be used, offering to developer’s abilities in their integration and mounting. This strategy is followed for the electrochemical sensing of cortisol [22,23]. However, the sensor requires an insightful understanding of electrochemistry and materials to be manufactured. Finally, they miss the portability feature because a PC connection is required.

In this paper, we contribute with the design and development of a portable, low-cost, customizable sensor platform, based on a potentiostat, and entirely made up of COTS components, including the sensor, in order to facilitate the reproducibility to end-users. Additionally, our platform includes a communication module to allow remote data acquisition and to facilitate the information reading for users and specialists located outside of the measurement site. The solution proposed here has been exhaustively validated with a well-known professional potentiostat [24]. We employ ascorbic acid as the substance under consideration, due to its extensive, well-documented record [25], and it is typically used for checking and verifying traditional methods and procedures. Our results reveal the appropriate operation of our system in terms of applicability, usability, and reliability.

The rest of this paper is organized as follows. Section 2 introduces the methodology employed in this research, and describes the main parts of the proposed device regarding system architecture and software structure. Then, the results obtained are presented in Section 3, as well as the validation of the device designed, by means of its calibration line and a repeatability study. Section 3 also explores the reusability of the screen-printed electrodes. Section 4 elaborates on the results discussion. Finally, Section 5 summarizes major conclusions.

## 2. Materials and Methods 

### 2.1. Reagents and Validation Instruments

All chemicals employed were of analytical grade and without any further processing. Sample solutions were prepared with 99% pure, solid state L-ascorbic acid (C_6_H_8_O_6_; 250 g), 95%–98% liquid sulfuric acid (H_2_SO_4_; 1000 mL), and MilliQ water (25 °C, Ω = 18.2 MΩ/cm). A set of PGSTAT100 Potentiostat/Galvanostat, from Metrohm-Autolab B.V. controlled by NOVA 1.11 electrochemical software [14] was used to validate the system proposed, due to its recognized robustness and reliability, compared to other commercial potentiostats.

### 2.2. Sample Preparation

Environmental conditions such as light, temperature, and humidity quickly degrade the ascorbic acid (AA), causing unwanted, uncontrolled, and irreversible processes. The resulting substance is an oxided form of AA, denoted as dehydroascorbic acid (DHA). Considering this chemical reaction, there are two basic approaches to quantify the AA into a substance, namely: (i) ignoring the possible presence of DHA, which negatively impacts on the measurement accuracy; and (ii) calculating the AA concentration as the sum of both AA and DHA, thus employing the HPLC technique. Concerning the latter, a specific method was proposed and developed in [26], where AA is deliberately oxidized to a more stable DHA substance using a sulfuric acid solution. Thereby, the DHA measurement obtained will correspond to the AA concentration. To this end, a set of AA aqueous solutions was prepared, using 0.5 M sulfuric acid as a solvent and a fresh solution for every experiment. In particular, sixteen different AA solutions, ranging 2 × 10*^−^*^3^–1 × 10*^−^*^6^ M were used as samples to study the functionality of our device. 

### 2.3. System Architecture

**Figure 1:**
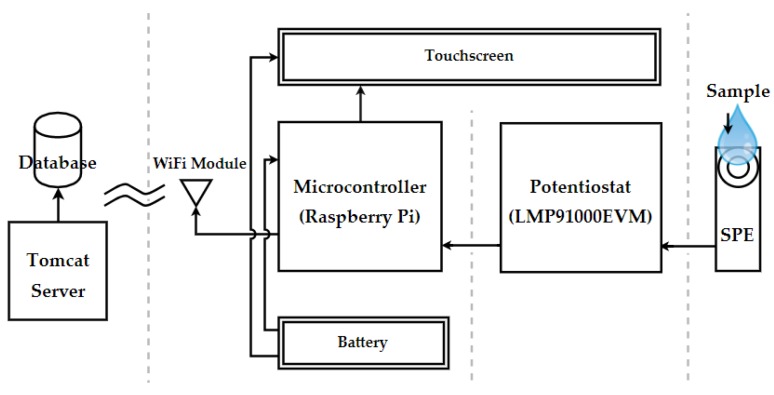
Hardware connections and data flowchart.

Figure 1 shows the system architecture of the device, designed, and developed to accurately determine the AA concentration in a solution through COTS components. The core consists of an LMP91000EVM potentiostat [27] regulated by a Raspberry Pi 2 Model B microcontroller (Raspberry Pi Foundation, Cambridge, UK) [28]. A commercial SPE from DropSens [29] (ref. DRP-110) may be inserted through an SPE adapter from IORodeo, which is connected to a handmade 2-mm jack interface. The system is powered by a lithium battery through a power pack expansion board (3800 mAh 5 V/1.8 A). This battery is directly connected to the microcontroller and a 10.1-inch HDMI LCD touchscreen, which facilitates the device setting and reading by means of a specific graphic user interface (GUI). Finally, a Wi-Fi module (Wireless N300 Nano USB Adapter) from D-Link is responsible for remotely dispatching the results obtained, which will also be stored in a database installed in a Tomcat server.

From a technological point of view, the process to determine the AA concentration through electrochemical methods based on an SPE is as follows. Firstly, a 50 µL drop of AA is poured over the carbon electrode surface to generate a potential difference between electrodes. It is important to cover the 3 carbon electrodes completely, and advisable to employ a suitable micropipette (in our case, a micropipette ranging 1–100 µL). Two electrodes are involved in this process: (i) the working electrode (WE), in which the redox reaction of the sample containing AA takes place, resulting in the electric current of interest calculating the concentration value; (ii) The counter electrode (CE) allows this current flow owing to a potential sweep applied to the WE related to the reference electrode. The applied potential originates the AA oxidation on the WE and the concomitant current flow between the WE and the CE as the experiment progresses. In the used SPE, from DropSens, both WE and CE are printed with carbon. The mentioned reference electrode (RE) is a non-polarized electrode keeping a constant potential (E_h_) and having a high entry impedance hindering any significant current flow through. In our case, RE is printed using silver in order to obtain these properties. It is required to control the potential applied to the WE. Secondly, the microcontroller forwards specific commands to the potentiostat to stimulate and control the analyte reaction that occurs in the WE of the SPE surface. The microcontroller also amplifies the generated current across the WE, to be processed later for the test. In our particular case, the stimulation process comprises a potential cycle, that is, a scan/sweep in electric potential (both direct and inverse) between two voltage limits. The described process is known as cyclic voltammetry (CV, henceforth), which will ease the relevant validation due to its popularity and implementation in the most of commercial potentiostats. Furthermore, the CV traces provides valuable information about reduction and oxidation phases, and thus the reversibility and irreversibility of the process. As a final step, a precise AA concentration is eventually obtained through a calibration line, as will be explained in more detail below.

Concerning the core of the device, and considering that open-source, low-cost, and high computing performance features should be satisfied, a Raspberry Pi 2 model B has been selected because of its built-in microcontroller and peripherals. The same procedure has been applied to choose the potentiostat. The resulting unit was the LMP91000 chip evaluation module (LMP91000EVM), which includes a chip with the same name. The LMP91000 is a programmable analog front-end (AFE) potentiostat, configured to perform CV measurements with 3-lead biosensors, making use of SPEs and the solution under test. An analog-to-digital converter (ADC161S626, Texas Instruments, Dallas, TX, USA) has also been integrated into the evaluation module, enabling the digitalization of the output voltage. This module is pre-designed to be attached through a GPSI16 connector to an SPIO4 board, which, operating with the required software, allows practitioners and users to obtain the CV results. However, this option was discarded due to the cost of the SPIO4 and the inability to check the state of the connections. In this way, it was decided to study how the Raspberry Pi and LMP91000EVM could be interfaced, using some female–female jumper wires. For this purpose, we configured the LMP91000 chip through the I^2^C protocol, and received the digitalized ADC output by means of the SPI protocol. The end connection scheme of the proposed interface is specified in Table 1. 

### 2.4. Software Description

**Figure 2:**
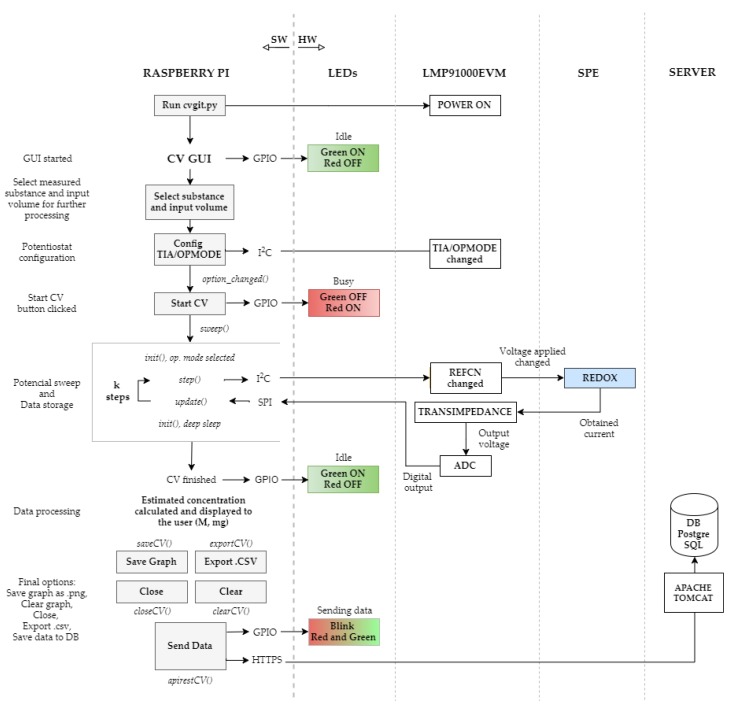
Functionality of hardware components and their associated software.

Figure 2 illustrates a complete diagram of the software structure. Commands and instructions are handled by the microcontroller through scripts that were written in Python programming language. The main script is denoted as cvgit.py, and is run each time its desktop shortcut is clicked, activating an intuitive and understandable GUI, programmed through Python’s TkInter widgets package. Simultaneously, GPIO channels, handled by the RPi. GPIO module in Python, turn on a green LED, indicating an idle status. This *cvgit.py* file loads, on the one hand, the *var.py* script, which contains all the variables, register addresses, and configuration parameters needed to enable SPI and I^2^C communications, and, on the other hand, the *settings.py* file, which encapsulates functionalities of these protocols, such as the acquisition and processing of data from the ADC.

**Figure 3:**
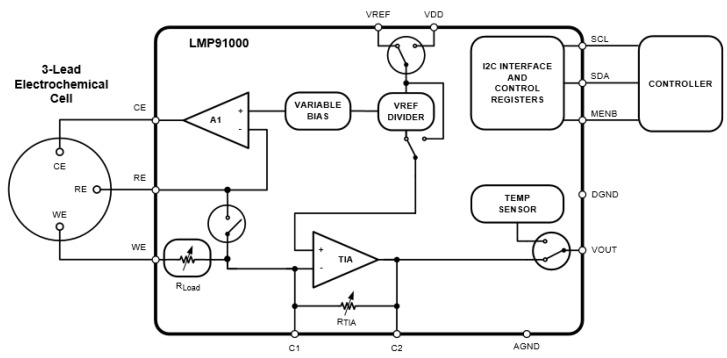
LMP91000 Simplified application schematic [30] (© Texas Instruments Incorporated).

Following the logical flow of the CV procedure, the configuration of the LMP91000EVM by means of the I^2^C *smbus* library is a key aspect to consider. In Figure 3, we observe that the LMP91000 chip consists mainly of two operational amplifiers, configurable voltage dividers/resistances, and a temperature sensor.

In detail, the first amplifier, called A1, connects to the RE and CE electrodes. This amplifier stabilizes the redox reaction occurring in these electrodes, while the potential sweep is applied. The sweep is performed through the *variable bias,* which allows the configuration of the voltage, divided into steps ranging from 0% to 24% of the V_REF_ (reference voltage). The LMP91000 datasheet [30] specifies that these steps are not regular, using steps of 1% until 2% of the V_REF_ is achieved, and then steps of 2% until 24% of the V_REF_, resulting in a total of 14 steps. Another issue to consider is the REFCN register, which is used to modify the *variable bias*, to tune the value of the voltage applied to the electrodes. This register also allows the setting up of the bias sign (positive or negative), the internal zero, that is, the voltage at the output of the LMP91000 chip (V_OUT_) when no reaction occurs in the electrodes, and to select the reference source used, external (from another device), or internal. R_LOAD_ is a fixed 10 Ω resistance connected to the working electrode. The second operational amplifier, called TIA, is responsible for converting the current obtained by the WE into voltage through a transimpedance R_TIA_, which may be configured by the user. Both R_TIA_ and R_LOAD_ resistances are controlled by the TIACN register exclusively dedicated to this purpose. Different values of transimpedance allow for the adjustment of the output current range. For instance, if the electric current level is very small, due to a much-diluted solution or a very low responsive sensor, a high R_TIA_ will be required to maintain the output voltage in the ADC range. On the contrary, a very high current level will result in a low R_TIA_ value.

According to the mentioned datasheet, different operation modes are possible. However, we only employ the 3-lead amperometric cell, temperature sensor, standby, and deep-sleep modes. The first mode allows to provide a complete CV procedure for 3-electrode sensors. The temperature sensor is used to check the suitable operation of the ADC software libraries; if the correct temperature is registered, the correct digitalization process work is assumed. The standby mode is the state set among scans to reduce power consumption. On the other hand, the deep-sleep state occurs when the chip is not performing any electrochemical activity during a long-time period. Note that both R_TIA_ and the operational modes may be changed by the end-user, via the developed GUI. In the event of successful modification, an acknowledgement message is displayed in the GUI console. Users can also configure other optional parameters such as (i) the desired title of the measurement, with which the program will save the .csv and .png files; (ii) the desired substance under test, in order to compute the molarity and weight through its corresponding calibration line (currently only AA calibration is available); and (iii) the volume required to perform the conversion from molarity to weight (mg). Once the GUI has been configured, to start a CV procedure, the end-user only needs to activate the start button of the available menu, switching the LED status of the device to busy (red). Then, the *sweep* function is executed. Besides the start and end configurations for the standby and deep-sleep states, this method incorporates two additional functionalities. On the one hand, the *sweep* function calls the *step* method, which, as described, modifies the REFCN register. On the other hand, it invokes the *update* function to acquire the digital output data from the ADC by means of the SPI protocol, which is managed by the *spidev* library. This data acquisition fulfills the ADC and LMP91000 specifications, in accordance with Equations (1) and (2) below,

V = ((V_RAW_·SPAN)/BR) + V_REF_ (V), (1)

I = ((V − (V_REF_/2))/R_TIA_) × 10^6^ (µA), (2)

where V_RAW_ is the ADC raw read voltage, SPAN is the full-scale voltage range calculated from (V_A_ − (V_REF_/2^16^)), with V_A_ as the single analog supply (5 V). BR is the maximum decimal value for an *n*-bit binary code, i.e., restricted to 16-bit resolution (as in our case), BR is 2^16^ − 1 = 65,635. Finally, the V_REF_ is set to 2.5 V to later perform the sweep of interest. To calculate the current, the Ohms law equation is applied, considering the resistance and voltage involved in the process. Both expressions, as well as their specific nomenclature, are taken as input parameters of the *update* function, which presents the results as a current/voltage plot on the GUI console, updating them as the CV sweep advances. To this end, the *update* function is supported by *matplotlib*, a plotting library for Python. The CV process conducts a complete sweep, ranging 0–0.6 V (24% of V_REF_) in steps of 50 mV per second, that is, steps of 2% of the V_REF_, as shown in Table 2. 

For this purpose, the REFCN register points to an external source as voltage reference, and sets 50% of the V_REF_ (1.25 V, since the V_REF_ is fixed at 2.5 V) as the internal zero. Under this configuration, for example, a potential difference of 0.3 V between WE and RE is regulated by 12% of the V_REF_ REFCN register value. Regarding the internal zero, when no electrochemical activity takes place, the output voltage is a fixed value of 1.25 V. In short, the step and update functions should be sequentially called on 25 occasions, 13 times from the direct sweep (0 V → 0.6 V), and 12 more times from the inverse sweep (0 V ← 0.6 V), thus avoiding measuring the 0.6 V point twice. A better resolution can be achieved by adding an external DAC able to provide multiple voltage values that acts as reference sources. This advanced configuration will be developed in the next versions of the device.

The CV procedure finishes when the sweep returns to zero. Then, functions such as save graph into a .*png* file (*saveCV*), save the data array into a *.csv* file (*exportCV*), clear graph (*clearCV*), close program (*closeCV*), or send data to a database will pop up in the GUI menu. It should also be noted that our device is able to remotely send information thanks to a Wi-Fi interface. In this sense, current and voltage data may be transmitted to a PC, which maintains a PostgreSQL database and an Apache Tomcat server to store and manage data results. Substance concentration under consideration may be calculated by interpolating the received current on a calibration line. This function was obtained by means of a linear regression of dataset (*x*, *y*) comprised by the maximum registered current (*y*-axis) in each resulting CV of a set of solutions with a well-known molarity (*x*-axis), both in logarithmic scales. Thereby, when an unknown molarity solution of AA is measured, only the regression expression is necessary to compute it. This feature is included in the software developed for the proposed device and is displayed to the end-user in the GUI. In other words, once the CV is performed, the molarity ‘M’ (mol/L) measured in the sample being tested and the corresponding weight ‘m’ (mg) related to an input volume are directly displayed on the GUI console. Molarity is obtained from calibration line, as explained above. Then, the weight (mg) is calculated by means of the relation among the molecular weight W of the substance, the input volume V (1 L by default), and the obtained molarity M, as is showed in Equation (3).

m (mg) **=** [M (mol/L) · V (L) · W (g/mol)] **/** 1000 (3)

**Figure 4:**
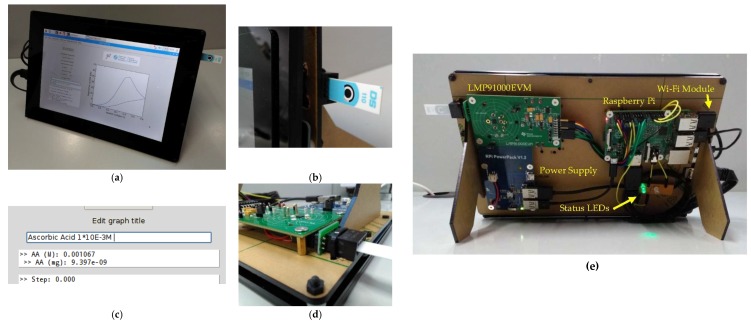
Final potentiostat prototype: (**a**) front view (with the application running on the screen); (**b**) detail of an inserted commercial Dropsens DS110 SPE; (**c**) zoom of an achieved concentration value a 1 × 10^−3^ M molarity; (**d**) rear view, focusing on the SPE plug and three electrode connections from the LMP91000EVM unit; and (**e**) general rear view of the entire device in an idle state (green LED turned on) with all components labeled.

Figure 4 shows the end prototype and its main hardware components. Both software and hardware documentation is available online [31] to facilitate the best project understanding and reproducibility to the audience.

## 3. Results

### 3.1. Calibration Line, Range and Reliability

**Figure 5:**
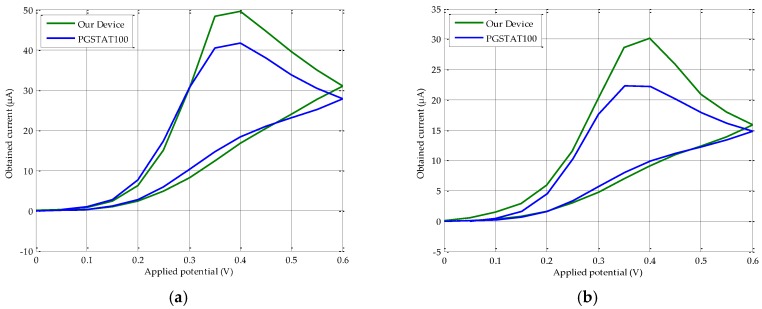

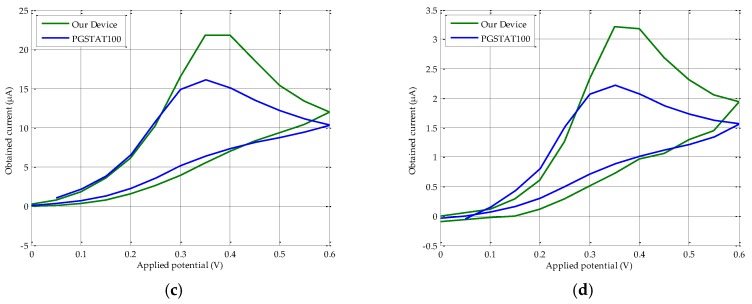
CV results comparative using commercial screen-printed electrodes (CE, WE and RE) between our device and PGSTAT100 for AA solutions of different molarity (**a**) 2 × 10^−3^ M; (**b**) 1 × 10^−3^ M; (**c**) 6 × 10^−4^ M; (**d**) 1 × 10^−4^ M.

**Figure 6:**
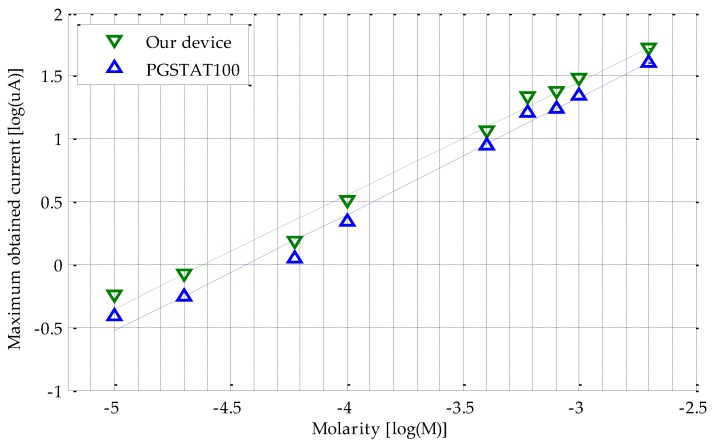
Calibration comparison between our device and the PGSTAT100 potentiostat.

A comparative study with a PGSTAT100 potentiostat was conducted to validate the proper operation of our device. The same adapter as that of the proposed device [32] has been used to connect the commercial screen-printed electrodes (CE, WE, and RE) with the corresponding PGSTAT100 jacks, by means of three two-millimeter jack clamps. The distinctive shape of the resulting CV curve should be similar in each sweep for both pieces of equipment. To this end, several CV were obtained for different AA concentrations, resulting in the plots depicted in Figure 5. As can be observed, the higher the molarity, the greater the registered current. From these results, we selected the maximum current value (peak) of each AA solution (commonly five to six values are enough, as indicated in chemical analysis basics [33]) to obtain the calibration curve, as shown in Figure 6. In particular, sixteen AA solutions were prepared. The most highly diluted among them presented a high signal to noise ratio. This is shown by a very unstable output current, due to many undesired peaks. In contrast, the most concentrated solutions led to ADC saturation. Therefore, only nine solutions were considered to have the appropriate behavior, that is, noise (too low molarities) or saturation (too high molarities) phenomena were not detected. These correctly responding solutions were used to further extract the peak current and draw the calibration curve. To increase the visibility of lower concentrations and currents, we decided to represent the results in a logarithmic scale. Following these premises, our device obeys a linear log–log relationship, according to the following expression:

log_10_(I(µA)) = 0.90 × log_10_(M(mol/L)) + 4.16, (4)

with an R^2^ = 0.988 regression coefficient. The same procedure was replicated in order to calibrate the PGSTAT100 unit; deriving Equation (5):

log_10_(I(µA)) = 0.93 × log_10_(M(mol/L)) + 4.11, (5)

with, in this case, the regression coefficient as R^2^ = 0.989. This means that our device operates on a wide concentration range, 10*^−^*^6^*–*10*^−^*^2^ M. Despite the clear parallelism between both calibration lines, the reliability of our device was also calculated. For this purpose, the sensitivity figure was compared, which related the slope of both lines in terms of the coefficient ((0.90/0.92) × 100). The result shows a reliability greater than 97%.

### 3.2. Repeatability

**Figure 7:**
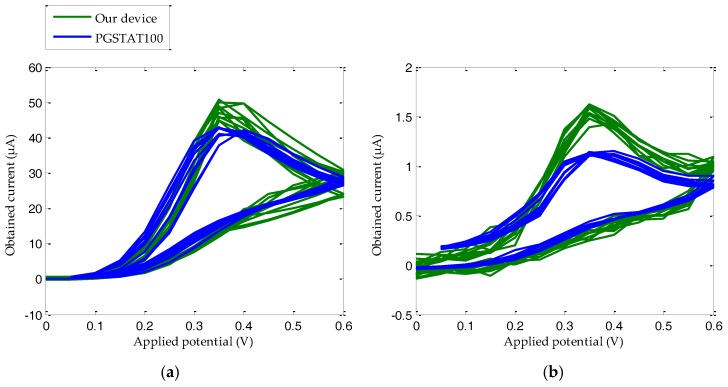
Device repeatability curves for ten samples of solutions of AA (**a**) 2 × 10^−^^3^ M and (**b**) 6 × 10^−^^5^ M.

Another important positive feature which characterizes and validates our device is repeatability. This term refers to how much a measurement changes in relation to a new one, repeating the test using the same type of SPE sensors (a new one for each measurement), solution samples, and, to the extent possible, under the same environmental conditions (temperature, humidity, pressure, etc.). It is well known that the set PGSTAT100 potentiostat and DS110 sensors ensure high repeatability (provided that appropriate storage, maintenance, and use are guaranteed). Therefore, our efforts were addressed to obtaining results as close as possible to this set. In practice, 2 × 10^−^^3^ M and 6 × 10^−^^5^ M solutions of AA were prepared and further measured with ten different DS110 sensors, each. These concentrations were selected to evaluate the repeatability at the ends of the calibration line, since intermediate values should also be reasonably compliant. Figure 7 shows ten superimposed CV curves measured for both devices. As expected, the current average value of our device is higher than the one measured with the PGSTAT100 potentiostat, with these data in accordance with the CV curves shown in Figure 5, above. Regarding our device, dispersion in respect to the average denoted as the relative standard deviation (RSD) is 4.60% and 4.27% for the electric current peaks corresponding to the CV curves for 2 × 10^−^^3^ M and 6 × 10^−^^5^ M respectively. On the other hand, RSD values of 1.81% and 1.20% were obtained with the PGSTAT100 equipment for 2 × 10^−^^3^ M and 6 × 10^−^^5^ M respectively. More details on the average and RSD values of the maximum electric currents are indicated in Table 3.

### 3.3. Sensor Reusability

**Figure 8:**
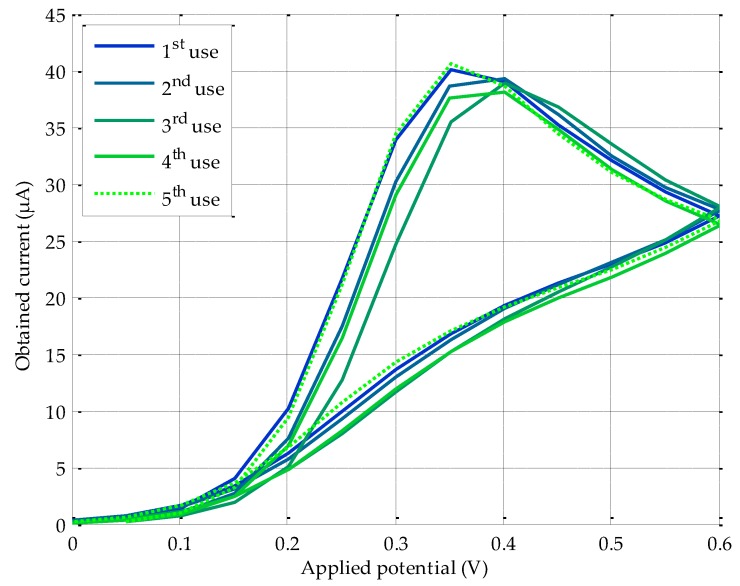
Repeatability study performed by means of a PGSTAT100. CV average for five consecutive uses and three different DS110 screen printed carbon electrodes.

Reusability is becoming an increasingly important metric in this specific scientific area, in which SPE sensors are consumables that are quickly used and discarded in each test, thus increasing the cost of the experiment. So, it is highly advisable to perform a reusability study, especially for applications where, on the one hand, a reduction of the cost factor is mandatory, and, on the other hand, commercial screen-printed carbon electrodes are subject to biochemical degradation when using them more than once. To this end, a battery of electric current measurements was carried out, operating a PGSTAT100 and implementing a straightforward procedure: the same SPE (DS110) sensor is immediately washed after the CV with MilliQ water, and its surface dried with a clean disposable paper towel to straightaway take a new measurement, extracting a sample of the same solution of AA (in this case, a 2 × 10^−3^ M solution). This process was repeated five times to estimate the sensor behavior after each additional re-use. To guarantee the reliability of the experiment, three different SPEs were employed, obtaining, for each use, the average of the three measurements, together with the deviation of this average with respect to the value obtained for the first use. These current outcomes are shown both in Table 4 and in Figure 8, and discussed in the next section.

## 4. Discussion

As can be observed in Figure 5, most of the electric current values measured with Autolab equipment match the ones obtained by our solution, except in the 0.4–0.6 V range of the direct sweep, where a significant difference is found. This deviation is basically produced by the differences in the electronic components employed in their respective developments. In particular, the transimpedance amplifier has a limited number of internal gain resistors. Under these circumstances, in comparison with the PGSTAT100, a lower adjustment to the entry voltage of the analog-to-digital converter (ADC) is reached. These particular points around 0.4–0.6 V coincide with those steps of the potential difference sweep where a higher current is generated. For instance, for a 1 × 10^−3^ M solution CV, the PGSTAT100 registers a maximum current of 22.24 µA, while our device measures 30.20 µA. A priori, this deviation is a drawback in terms of accuracy. However, analyzing the calibration curves (see Figure 6), the results reveal that the maximum difference of current between the PGSTAT100 outcomes and our potentiostat development for each CV remains constant. An average difference of 28.17% between the maximum obtained currents in PGSTAT100 and in our device, is experienced for the entire studied range. Another fact worth noting when operating with electrochemical-based sensors, is the slightly growing variability in low concentration solutions, in comparison with the higher ones for the CV conducted in the same way (Figure 5 and Figure 7). In any case, an optimal fitting with a regression coefficient higher than the 0.95 standard is achieved. This value, together with a reliability of around 97% between calibration plots, guarantees the excellent proportionality between both platforms, which in turn, becomes a “quasi” constant offset. Therefore, from these parallel calibration lines, the following two facts can be inferred:

1) Our calibration curve is thoroughly valid for interpolating values different from the proposed ones in this work. That is, if the maximum current measured is 0.5 μA, a molarity of 1.13 × 10^−4^ M is reached, as shown in Figure 6.
2) The current peaks obtained for the PGSTAT100 and our device can be introduced in Equations (3) and (4) to derive the same molarity.

To sum up, the results achieved in this work show that our potentiostat implementation is comparable to the costly Autolab machine. This affirmation is further verified when analyzing the repeatability results. As could be expected, our device has a greater RSD than the PGSTAT100 potentiostat. However, these values differ by less than 5% (µA), which translates into good performance by our device. 

Some interesting considerations can be also drawn from the reusability study (see Figure 8). Taking into consideration the calibration plot for our solution, modifying the value of the generated current in 1 μA involves a change of 2.45 × 10^−4^ M in the molarity of the measured substance. Therefore, depending on the application under study and target sensitivity, a single DS110 SPE can be reused (four times for an AA solution measurement), which results in an additional reduction in consumable cost. For instance, three uses of the same sensor lead, approximately to an average deviation (error) with respect to the first use of 1.88 µA, which in molarity means: 1.88 µA × 2.45 × 10^−4^ M/µA = 4.61 × 10^−4^ M. This value can be negligible or significant, depending on the specific application and substance to be measured, since it will have an impact on the SPE modification, environmental conditions, required accuracy, etc. From the fourth and subsequent re-uses, the registered current increases, giving rise to undesirable effects, such as carbon surface distortion in the SPE, therefore, jeopardizing reliability.

## 5. Conclusions

The main contribution of this paper is the design and development of a portable, electrochemical-based solution to accurately measure the AA concentration outside of the usual laboratory environment. Therefore, our proposal is suitable for outdoor measurement campaigns, enabling new perspectives and innovative applications in yet unexplored fields (e.g., to know the level of AA concentration in fruit and vegetables, directly in the field). The system designed satisfies the following three aspects: (i) it is disposable and cheap, and unmodified general-purpose screen carbon printed electrodes may be employed as sensors; (ii) it is made from commercial off-the-shelf (COTS) components, which can be easily acquired and integrated, providing portability and low-cost; and (iii) it incorporates communications to transmit acquired data to remote ends. In addition to the hardware system integration, robust software to control the entire device and the sensor measurements has also been developed. Raw acquired data are further processed to extract useful information that is presented to the end-user. For this purpose, specific GUI-controlled software is programmed as a means of providing seamless interaction between the device and the end-user (e.g., molarity in milligrams and number of moles are directly calculated from the CV). Finally, the device is able to connect to a remote database to store all the acquired information, ready for future queries and for automated big data analysis. 

A well-known, standard, but costly, laboratory potentiostat, such as the PGSTAT100, has been employed to validate the functionality of our device. To this end, a thorough performance comparison between both platforms has been accomplished. Results obtained reveal the positive performance of our proposal in terms of CV curves and repeatability. As an additional contribution, a reusability study was carried out, indicating a potential cost reduction in consumable supplies, if several consecutive measurements are allowed for the same screen carbon printed electrode. However, this will finally depend on the specific application and required accuracy. At this moment, only AA was considered as a test substance, our purpose being to broaden the scope in the future, increasing the number of target substances. The CV approach presented here is observed as a solid base to analyze other substances. This will only require an adequate SPE sensor type for the new substance under consideration, and minor changes to the device (to improve CV dynamic modification of the settings and parameters and extend the system to additional amperometric techniques), highlighting the versatility of the system proposed.

## Figures and Tables

**Table 1:** Physical pin numbers used to interface Raspberry Pi and LMP91000EVM.

Raspberry Pi	Connection Name	Protocol	LMP91000EVM
19	MOSI	SPI	7
21	MISO	SPI	5
23	SCLK	SPI	3
24	CE0	SPI	1
3	SDA	I^2^C	11
5	SCL	I^2^C	12
9, 14, 6, 39 *	GND *	-	2, 4, 8, 10 *
2	5V	-	14
1	3.3V	-	13

* Order is not relevant in ground pins. Pins not shown might be open.

**Table 2:** Procedure of the potential sweep performed. Percentage of the reference source applied, the equivalent voltage, and the binary code necessary to configure the REFCN register.

**% of V_REF_**	0	2	4	6	8	10	12	14	16	18	20	22	24
**Potential Step (V)**	0.00	0.05	0.10	0.15	0.20	0.25	0.30	0.35	0.40	0.45	0.50	0.55	0.60
**REFCN Register (Binary)**	10110000	10110010	10110011	10110100	10110101	10110110	10110111	10111000	10111001	10111010	10111011	10111100	10111101

**Table 3:** Repeatability values between PGSTAT100 and our device.

Molarity (M)	PGSTAT100 x̄ (µA) ± RSD (%)	Our Device x̄ (µA) ± RSD (%)
2 × 10^−3^	41.99 µA ± 1.81%	47.51 µA ± 4.60%
6 × 10^−5^	1.12 µA ± 1.20%	1.55 µA ± 4.26%

**Table 4:** Reusability study of DS110 SPE.

Uses	Max. Current (µA)	Deviation in Respect to the 1st Use (µA)
1st	40.02	0
2nd	39.29	0.73
3rd	38.93	1.09
4th	38.14	1.88
5th	40.62	0.60
